# The Co-Occurrence of Autism and Attention Deficit Hyperactivity Disorder in Children – What Do We Know?

**DOI:** 10.3389/fnhum.2014.00268

**Published:** 2014-04-29

**Authors:** Yael Leitner

**Affiliations:** ^1^Child Development Center, Dana-Dwek Children’s Hospital, Tel Aviv Sourasky Medical Center, Tel Aviv, Israel; ^2^Sackler School of Medicine, Tel Aviv University, Tel Aviv, Israel

**Keywords:** autistic spectrum disorders, attention deficit hyperactivity disorder, diagnostic and statistical manual, co-morbidity, co-occurrence

## Abstract

Symptoms of attention deficit hyperactivity disorder (ADHD) and autistic spectrum disorder (ASD) often co-occur. The DSM-IV had specified that an ASD diagnosis is an exclusion criterion for ADHD, thereby limiting research of this common clinical co-occurrence. As neurodevelopmental disorders, both ASD and ADHD share some phenotypic similarities, but are characterized by distinct diagnostic criteria. The present review will examine the frequency and implications of this clinical co-occurrence in children, with an emphasis on the available data regarding pre-school age. The review will highlight possible etiologies explaining it, and suggest future research directions necessary to enhance our understanding of both etiology and therapeutic interventions, in light of the new DSM-V criteria, allowing for a dual diagnosis.

## Introduction

In the last decade, studies have reported increased prevalence of both attention deficit hyperactivity disorder (ADHD) and autistic spectrum disorders (ASD). While ADHD is defined by impaired functioning in the areas of attention, hyperactivity, and impulsivity, whereas ASD is characterized by core social dysfunction and restrictive-repetitive behaviors, studies show that between 30 and 50% of individuals with ASD manifest ADHD symptoms (particularly at pre-school age), and similarly, estimates suggest two-thirds of individuals with ADHD show features of ASD (Davis and Kollins, 2012). Recent findings from the Autism Treatment Network (ATN) database suggest that co-occurrence of ADHD and ASD is associated with a lower quality of life and poorer adaptive functioning than in any one of these conditions (Vora and Sikora, 2011). Both disorders often include difficulties in attention, communication with peers, impulsivity, and various degrees of restlessness or hyperactivity. Both are more common in boys than in girls, and present, at least partially, at pre-school age. Both disorders have a known genetic pre-disposition, with comorbidity within the same individual and across family members, and both syndromes cause significant behavioral, academic, emotional, and adaptive problems in school, at home, and elsewhere. (Rao and Landa, 2013).

Evidence for common neurobiological substrates has been found through similarities in neuropsychological profiles in individuals with both disorders (Gargaro et al., 2011; Rommelse et al., 2011). It has been shown that children with ADHD have pragmatic language difficulties similar to children in the ASD spectrum (Bishop and Baird, 2001). Further neuropsychological similarities are suggested by a study of emotional recognition and theory of mind, which showed that children with ADHD could not be distinguished from those with ASD (Buitelaar et al., 1999). A study on social perspective taking showed children with ADHD used lower levels of social perspective taking coordination in their definition of problems, identification of feelings, and evaluation of outcomes than children without ADHD, and these differences persisted after the role of language abilities, intelligence, and oppositional and conduct problems were taken into account (Marton et al., 2009).

Despite the growing body of research pointing at the frequent co-occurrence of these two disorders, the previous DSM-IV-TR has not allowed a dual diagnosis. The DSM-V, in its revised ADHD diagnostic criteria, recognizes the frequency of this co-occurrence and allows, for the first time, a co-morbid diagnosis of ADHD with autism spectrum disorder. This new attitude will not only allow for more efficient clinical management of these children, but will also clear the way for a more precise scientific understanding of the overlap of these two disorders. While most research to date has documented developmental trajectories for ADHD and ASD separately, little is known regarding their co-occurrence, particularly at young pre-school age. PUBMED was searched using the definition “co-occurrence of ASD and ADHD in pre-school children.” This led to only 35 studies, and therefore search was broadened to include the more general definition of the “co-occurrence of ASD (or social-communication difficulties) and ADHD in children.” More than 150 articles were eventually reviewed.

## ADHD (Symptoms) in Children with ASD

A significant percentage of children with ASD seeking services at clinical centers present with comorbid symptoms of ADHD, with rates ranging between 37% (Gadow et al., 2006) and 85% (Lee and Ousley, 2006) across studies conducted in the United States and Europe (Rao and Landa, 2013). ADHD was the third most common disorder identified in a community sample of 5–17 years old children (Leyfer et al., 2006), with 31% of the sample meeting full ADHD criteria and another 24% with subsyndromal ADHD symptoms. This is lower than reported rates of ASD and ADHD in clinic samples (Rao and Landa, 2013). Very few studies have looked at the epidemiology of co-existing disorders in pre-school age children diagnosed with ASD. Two year-old twins (*n* = 312) from the Boston University Twin project were studied by Ronald et al. (2010) for autistic-like traits and ADHD behaviors using Child Behavior Checklist (CBCL) answered by their parents. Controlling for cognitive abilities and socioeconomic status, autistic like traits correlated positively with ADHD behaviors (*r* = 0.23–0.26), a lower correlation than described for older children. In a recent survey by Carlsson et al. (2013), 198 pre-school Swedish children (age 4.5–6.5 years) with ASD who treated in a habilitation center, were assessed for such disorders. They found language problems in 78%, intellectual disability in 49%, below average motor function in 37%, and severe hyperactivity in 33%.

Lecavalier et al. (2009) has lately shown the validity of DSM-IV syndromes in a group of 229 pre-schoolers with ASD, using confirmatory factor analysis (CFA). Despite very high factor loading with the general ADHD combined factor, several items had non-significant loadings with the specific ADHD hyperactive–impulsive factor. In other words, the verbal items from the hyperactive–impulsive sub scale were negatively correlated with the other motor items suggesting hyperactivity–impulsivity has two components: physical and verbal. The authors comment that this is a good example of how the clinical features of ASD might alter the clinical presentation of a co-occurring DSM-IV defined (pre-school) ADHD. In a study by Sikora (Sikora et al., 2012), as part of the Autism Speaks ATN, data was collected from 14 sites in the US and Canada. Participants between the age of 2 and 17.9 years were included if they met DSM-IV and/or Autism Diagnostic Intervention Schedule (ADOS) diagnostic criteria for ASD, if they were cared for at an established ATN site, if parents was fluent in English, and it was the language spoken with the child at least 75% of the time. Parents were asked to complete the CBCL and the pediatric quality of life inventory (PedsQL) and were also interviewed to complete the Vineland Adaptive Behavior Scales (VABS-II). Cognitive scores were used as covariates. Of the whole study group, 1737 participants were 2–5 years old. Of these, 40% had elevated T score in 1 of the 2 ADHD-related scales, and 18.8% had both ADHD-related scales T scores ( >70) elevated. The ASD + ADHD group had lower scores on both the VABS-II and the PedsQL. The authors conclude that over one third of children with ASD have some comorbid ADHD symptoms, and their presence is related to greater problems in adaptive skills and poorer overall quality of life, and suggest primary care providers should screen for symptoms of ADHD in their patients with ASD, and consider these symptoms when developing a care plan.

## ASD Symptoms (Social-Communication Difficulties) in Children with ADHD

Social problems are not part of the core diagnostic criteria for ADHD, but children with ADHD experience significant social difficulties (Cantwell, 1996; Friedman et al., 2003). ADHD children are more often rejected by their peers, and have fewer friends (Hoza et al., 2005; Mikami, 2010). In many cases, these difficulties are viewed as a direct result of the ADHD core symptoms. Inattentive behaviors may lead a child to miss social cues, impulsiveness may result in upsetting peers, and hyperactivity hinders participation in organized activities and leads to avoidance of peers. It is estimated that approximately 50–60% of ADHD children experience rejection by their peers (Barkley et al., 1990). In fact, many ADHD children are disliked within minutes of the initial social interaction (Pelham et al., 1985) and then denied further opportunities to practice social skills, which lead to further rejection (Milich et al., 1982). Specific play behaviors have been linked with rejection of ADHD children and include being bossy, intrusive, inflexible, controlling, annoying, explosive, argumentative, easily frustrated, inattentive during organized sports/games, and violating the rules of the game (Pelham et al., 1985; Whalen et al., 1985; Klassen et al., 2004; Young et al., 2005). Social functioning by ADHD subtype varies somewhat according to rater (e.g., teachers, parents, and peers); however, the general consensus is that all ADHD subtypes are at risk for peer rejection (Carlson and Mann, 2000; Hodgens et al., 2000). The presence of co-morbid psychiatric disorders tends to exacerbate social impairments in children with ADHD (Greene et al., 1996; Antshel and Remer, 2003). This is significant when considering that over 2/3 of individuals with ADHD have a co-morbid psychiatric disorder (Cantwell, 1996) with rates reported to be 15–75% with mood disorder, 25% with anxiety, and 30–50% with conduct disorder (CD). Others (Biederman et al., 1991; Eiraldi et al., 2000) have found that anxiety and depression together accounted for 30% of the variance in social impairment in ADHD. Children with both ADHD and a learning disability have also been found to have greater peer relations difficulties than children with only a learning disability (Flicek, 1992).

This profile of social difficulties differs, however, from that observed in ASD, in which impairment in the basic understanding of social realm is central, such as a lack of emotional reciprocity and engagement with others, and the low interest or enjoyment in social interaction. Cantwell (1996) described a type of social difficulty in ADHD by a “lack of savoir faire,” and estimated that this social naivety may affect some 20% of ADHD children and adolescents. Recent research suggests that many individuals with ADHD may experience social impairments that are more consistent with those observed in ASD. In children with a primary diagnosis of ADHD, the level of autistic symptomatology corresponded to the severity of ADHD subtype; children with the combined type of ADHD-demonstrated the most autistic symptoms (Reiersen et al., 2007). In a study recently published by Kotte et al. (2013), a positive autistic traits (AT) profile operationalized from the CBCL, was significantly overrepresented among ADHD children vs. controls (18 vs. 0.87%; *P* < 0.001).

Recent research suggests many individuals with ADHD may experience social impairments consistent with those observed in ASD. Santosh and Mijovic (2004) characterized the social impairments in children with ADHD as associated with either relationship difficulty (conduct and affective problems), or with social-communication difficulty. Children with the latter were more likely to exhibit repetitive behaviors, speech and language impairment, and developmental problems similar to ASD. Other investigators have described deficient empathy and facial affect recognition in children with ADHD (Sinzig et al., 2008; Uekermann et al., 2010). Other studies have also pointed at the increased rate of autistic symptoms in samples of children with ADHD. Grzadzinski et al. (2011) confirmed the presence of a sub group of children with ADHD and elevated ratings of core ASD traits not accounted for by ADHD or behavioral symptoms. The ADHD group with AT revealed greater ODD behaviors than those with ADHD-only. Most of the studies conducted in middle or late childhood have shown that a substantial proportion of children with ADHD show significant autistic symptoms (Santosh and Mijovic, 2004; Holtmann et al., 2007; Nijmeijer et al., 2008). Very few studies have looked at pre-school children with ADHD, in an effort to identify early “comorbid” ASD symptoms, perhaps because an ASD diagnosis is usually made at pre-school age, while a primary ADHD diagnosis is often delayed to late pre-school or early school age.

## Impact of Comorbid ADHD and ASD

Diagnostic constraints have limited research on co-occurring ADHD and ASD, because many studies, in accordance with the DSM-IV, have excluded individuals with other psychiatric or developmental difficulties (Davis and Kollins, 2012). There is preliminary evidence that when ADHD is comorbid with ASD, the risk for increased severity of psychosocial problems increases (Gadow et al., 2004; Yerys et al., 2009). Research comparing individuals with both diagnoses to individuals with a single diagnosis suggest that co-occurring symptoms are associated with greater impairment than a single diagnosis. By both parent and teacher reports children with ADHD and ASD (Rao and Landa, 2013) experience more difficulty in daily life. Furthermore, these co-occurring conditions may be less responsive to standard treatments for either disorder. Children 4–8 years old with ASD, whose parents report significant symptoms of ADHD, show lower cognitive functioning, more severe social impairment, and greater delays in adaptive functioning than children with ASD-only.

Sikora et al. as part of the activities of the ATN (Sikora et al., 2012) collected data on children with ASD across 14 sites in the US and Canada. Children were between ages 2 and 17.9 years. They were divided into groups based on whether their parents rated them as having clinically significant scores on ADHD problems subscales from the CBCL. 56.6% of the sample was young children between 2 and 5 years. Analysis revealed that those with ASD+ADHD symptoms had lower scores in all the areas measured. Psychosocial health summary, school functioning, physical functioning, emotional, and social functioning scores were all lower than those of the children with ASD alone (*P* < 0.0001). In another study by Gadow et al. (2004), PDD and non-PDD clinic groups showed equally severe ADHD and oppositional defiant disorder symptoms. As measured by parent and teacher referenced rating scale (ECI-4), Mulligan et al. (2009) compared autism symptoms in 821 ADHD probands, 1050 siblings, and 149 controls by using the Social-Communication Questionnaire (SCQ). Latent class analysis yielded five classes; class 1 (31%) had very few autism symptoms and low comorbidity; classes 2–4 were intermediate; class 5 (7%) had high autism symptoms and comorbidity. The cluster with the highest mean SCQ score (class 5) had the highest prevalence of co-morbid oppositional defiant disorder, CD, language disorder, and motor disorder. Other evidence supports more ODD symptoms in children with both disorders when using teacher rating rather than parent ratings (Guttmann-Steinmetz et al., 2009). These findings, however, describe mostly school age children, while there is a lack of psychiatric comorbidity studies in younger children with both conditions.

Several investigators have found (Sinzig et al., 2008; Rao and Landa, 2013) a higher percentage of children with both ASD+ADHD were classified as having significant cognitive delays than children with ASD-only (61 vs. 25%). Kotte et al. (2013) had reported ADHD children with high score on the AT profile of the CBCL, were significantly more impaired than control subjects in psychopathology, interpersonal, school, family, and cognitive domains. Yerys et al. (2009) reported on elevated rates of externalizing problem behaviors as well as greater impairment in executive functioning in children with comorbid ASD and ADHD, compared with children with ASD-only. Children with high functioning autism (HFA) and attention problems scored significantly below the children with HFA only, on the verbal memory and delayed recall measures, supporting the proposition that children with both disorders differ not only on a clinical level but also on a neurocognitive level (Andersen et al., 2013).

Only one study has looked at the relationship between the two disorders as they develop over time. St Pourcain et al. (2011) have followed 5,383 singletons (the Avon Longitudinal Study of Parents and Children-ALSPAC) ages 4–17 years and assessed multiple measures of hyperactive–inattentive traits and autistic social-communication impairment at multiple time points. Autistic symptoms were more stable than those of ADHD behaviors, which showed greater variability. Trajectories for both traits were strongly, but not reciprocally interlinked, such that the majority of children with a persistent hyperactive–inattentive symptomatology also showed persistent social-communication deficits, but not vice versa. Shared predictors, especially for trajectories of persistent impairment were maternal smoking during the first trimester, which included familial effects and a teenage pregnancy. The authors conclude that patterns of association between ASD and ADHD symptoms change over time, and propose to remove exclusivity criteria for these diagnoses in the DSM-V, as has already been done.

## Possible Etiologies for the Co-Occurrence of ASD and ADHD

Due to the previous DSM-IV diagnostic constraints, research concerning the possible etiologies of the co-occurrence of ADHD and ASD is scarce. The central focus of available research is in the fields of neuropsychology, genetics, and neuroimaging.

Although there are some important differences between the two disorders, as mentioned in the introduction, ASD and ADHD share many similar impairments in developmental and cognitive domains. Both are more common in males, have a strong comorbidity with intellectual disability, and are also associated with other specific learning and developmental difficulties, notably language, reading, and motor problems. Executive functions (EF) deficits are common in both disorders, together with response inhibition deficit. EF measures hardly discriminated between ADHD and HFA, but compared to children with ADHD, the HFA group showed more difficulty with cognitive flexibility and planning (Cooper et al., 2014). Children with ADHD have pragmatic language difficulties similar to children in the ASD spectrum (Bishop and Baird, 2001). Further neuropsychological similarities are suggested by a study of emotional recognition and theory of mind which showed that children with ADHD could not be distinguished from those with ASD (Buitelaar et al., 1999).

Rommelse et al. (2010) suggest a variety of hypotheses to explain co-occurrence, but the two most likely explanations may be that the two are independent disorders occurring together by association with a third independent factor, or alternatively they share a common underlying etiology. The authors believe the latter is the most likely model and that both disorders share a common genetic basis. Their view is supported by several family, twin, and molecular genetic studies. Both family (Holtmann et al., 2007; Guttmann-Steinmetz et al., 2009) and twin studies (Reiersen et al., 2007; Ronald et al., 2010) provide support for the hypothesis that ADHD and ASD originate from partly similar familial/genetic factors. About 50–72% of the contributing genetic factors in both disorders show overlap. These shared genetic and neurobiological underpinnings form an explanation why both disorders occur so frequently within the same patient and family.

Two related family studies originating from the IMAGE (Holtmann et al., 2007; Guttmann-Steinmetz et al., 2009) cohort have examined the rates and severity of ASD in probands with ADHD and their siblings. Mulligan et al. (2009) measured autism symptoms using the SCQ and compared 821 ADHD probands, 1050 siblings, and 149 controls. Affected and unaffected male (but not female) siblings had higher ASQ scores than controls. The phenotypic correlation between ASD and ADHD was slightly higher for males (0.63) than for females (0.49). Using a modified method of the deFries–Fulker analysis, the authors conclude that 56% of the cross-correlation ( =0.18), could be explained by shared genetic influences on ADHD and ASD. In another study by Nijmeijer et al. (2008), using the same cohort, 256 sibling pairs and 147 controls were studied, using the Children Social Behavior Questionnaire (CSBQ). This instrument measures less severe variants of ASD. Similar to Mulligan’s study, sibling correlations for ASD were significant, but contrary to the earlier study, correlations were higher in female probands (0.44), than male probands (0.23). Also, siblings correlations for ASD were higher in older than younger children, indicating an increased genetic influence on ASD within ADHD families over time. In this study, cross correlations were not significant, suggesting independent familial factors give rise to ADHD and ASD.

In a twin study, mentioned earlier (Reiersen et al., 2007), AT were examined in 8 years old ADHD twins (*N* = 6771), using parent and teacher questionnaires. Phenotypic correlations were 0.54 for parent and 0.51 for teacher data. The authors concluded that genetic correlations between ASD and ADHD symptoms were all >0.50. Similar results were reported in a study of young adult twins (*N* = 674), using a self reported measures of ADHD and ASD. A bivariate model indicated that the genetic correlation between ADHD and ASD was 0.72. Models with additive genetic effects and unique environmental effects fitted the data best, with no evidence for sex differences. While the limitation of most of these studies is the use of rating scales rather than direct clinical measures to evaluate either ADHD or ASD, together they do point at a genetic correlation between the two diagnoses.

Linkage studies and Genome Wide Association Studies (GWAS) have specifically addressed this co-occurrence, pinpointing to some promising pleiotropic genes, loci, and single nucleotide polymorphisms (SNPs). Their authors comment that there is an urgent need for better designed and powered studies to tackle this complex issue. A recent study (Cross-Disorder Group of the Psychiatric Genomics Consortium, 2013) using GWAS data from the Psychiatric Genomic Consortium (PGC), for cases and controls in five psychiatric disorders including ADHD and ASD, did not find any significant genetic correlation between ASD and ADHD, using common SNP’s. Analyses revealed that there were modest shared genetic influences between ADHD and AT as well as some common environmental influences explaining their co-variability. The contribution of assortative mating and parent-of-origin effects to the co-occurrence of ASD and ADHD has been investigated by van Steijn et al. (2012) in 121 families with at least one child diagnosed with ASD, and one or more biological sibling. All children and parents were carefully screened for the presence of ASD and ADHD. The authors concluded that cross-assortative mating for ASD or ADHD does not explain the frequent co-occurrence of these disorders within families. They did show, however, that parental ADHD is predictive of offspring’ ASD but not vice versa, hinting that risk factors underlying ASD may overlap to a larger degree with risk factors underlying ADHD than vice versa.

In autism, as opposed to ADHD, anatomical studies found larger total brain and white matter volumes in most cortical brain regions and in the cerebellum, caudate, and globus pallidum (Piven et al., 1996). A shared anatomical dysmorphology between the two disorders appears to be a smaller corpus callosum. In functional neuroimaging, the most consistent findings has been that of reduced frontal and parietal activation across a wide range of tasks in ASD (Baron-Cohen et al., 1999), which may not be different for ADHD. Geurts et al. (2013) tested whether there is a relationship between gray matter (GM) volume and autism and ADHD symptom severity by using structural MRI. They found that self-reports on symptom severity of both disorders correlated with GM volume in the left inferior frontal gyrus, but each disorder symptom severity was correlated separately with different cortical areas. They conclude it seems to be an oversimplification to typify psychiatric disorders solely as extremes of brain structure abnormalities. Di Martino et al. (2013) used voxel wise network centrality, functional connectivity metrics indexing local [degree centrality (DC)] and global (eigenvector centrality) functional relationships across the entire brain connectome, in resting state functional magnetic resonance imaging data from 56 children with ASD, 45 children with ADHD, and 50 typically developing children. In both clinical groups, cortical and sub cortical areas exhibited centrality abnormalities, some common to both disorders (in the precuneus), but others were disorder-specific. Secondary analyses differentiating children with ASD into those with or without ADHD-like comorbidity [ASD(+) and ASD(−), respectively] revealed that the ASD(+) group shared ADHD-specific abnormalities in basal ganglia. By contrast, centrality increases in temporolimbic areas characterized children with ASD regardless of ADHD-like comorbidity. The authors conclude that their work provides evidence for both shared and distinct underlying mechanisms at the large-scale network level. These and other neuroimaging studies in the future might lead to better understanding of the neuro-circuitry involved in the co-occurrence of ASD and ADHD.

## Treatment of Co-Occurring ADHD and ASD

Much evidence supports the use of medications to treat symptoms of ADHD in typically developing school age children (Subcommittee on Attention Deficit/Hyperactivity Disorder et al., 2011). ADHD medications have shown their efficacy in pre-school children (PATS study), but with less efficacy and more side effects than in school age children (Riddle et al., 2013). For ASD, medications target comorbid behavioral symptoms like irritability, hyperactivity, and aggression, rather than the core social and communication deficits. Several medications have demonstrated the potential to treat repetitive/stereotyped behaviors, but efficacy data has not been strong. Only two medications, to date, have been formally approved for use in ASD, and they both target irritability; Risperidone and Aripiprazole (Abilify) (Owen et al., 2009; Canitano and Scandurra, 2011; Williams et al., 2013). Despite limited research on the pharmacological treatment in ASD, there is a significant increase in the use of psychoactive medications in this group in recent years, in part due to an increase in the use in ADHD medications in ASD children with ADHD symptoms. Frazier et al. (2011) have recently used data on psychotropic medication from the first wave of the National Longitudinal Transition Study 2, a nationally representative study of adolescents ages 13–17 in special education, and have shown that youths with ASD+ADHD had the highest rates of use (58.2%), followed by youths with ADHD-only (49.0%) and youths with ASD-only (34.3%). Youths with ASD, both ASD-only and ASD+ADHD, used medications across a variety of medication classes, whereas stimulants were dominant among youths with ADHD-only.

## Stimulant Medications

Methylphenidate and atomoxetine (Frazier et al., 2011) are both typically used to treat ADHD and are also effective in ASD. Santosh et al. (2006) conducted a retrospective and an open-label prospective trial to compare response to stimulants (methylphenidate or dextroamphetamine) between children with ASD and ADHD and children with ADHD alone, and found no significant differences in treatment response or side-effect profiles between groups. However, other studies suggest that response rates of methylphenidate may differ in ASD as compared to what is reported in typically developing children with ADHD alone. The National Institute of Mental Health Collaborative Multisite Multimodal Treatment Study of Children with ADHD (MTA) (MTA Cooperative Group, 2004) reported response rates of 70–80% as compared to the 49% reported in the Research Units of Pediatric Psychopharmacology (RUPP) Autism Network trial of methylphenidate (Arnold et al., 2012). In terms of tolerability, 18% of subjects in the RUPP trial withdrew, yet discontinuation rates were quite low in the MTA study (1.4%). While methylphenidate may improve irritability in ADHD without ASD, it appears to worsen irritability in some patients with ASD.

Reduction in ADHD symptoms has been the primary outcome measure of most studies of co-occurring ADHD and ASD. A secondary analysis of the RUPP evaluated the effects of methylphenidate on social-communication skills and self-regulation in 33 children with ASD. Weekly dedicated observations over a 4-week period, indicated that methylphenidate use was associated with several positive social outcomes; including improved initiation of joint attention, improved response to bids for joint attention, and better affective and self-regulation.

## Non-Stimulant Medications

In the only controlled study of atomoxetine (Jahromi et al., 2009), results were significantly better than placebo, but the sample size was small and only 7 of 16 children (43%) were considered responders. Overall, both methylphenidate and atomoxetine appear to effectively treat ADHD-related symptoms in ASD (MTA Cooperative Group, 2004; Arnold et al., 2006, 2012; Posey et al., 2006; Santosh et al., 2006; Jahromi et al., 2009; Murray, 2010), but atomoxetine demonstrated better tolerability than stimulants in individuals with co-occurring ADHD and ASD. Response rates may, however, be lower in ASD plus ADHD, than in ADHD alone, and symptoms of inattention may be less likely to respond than symptoms of hyperactivity and impulsivity. Atomoxetine effectiveness may vary as a function of level of impairment, measured by cognitive ability or by ASD symptom severity (Arnold et al., 2006; Posey et al., 2006). Finally, treatment success may be limited by tolerability. Many studies have demonstrated efficacy for antipsychotics, and since the RUPP trial with risperidone this medication in particular has consistently shown benefit for hyperactivity in ASD (Murray, 2010; Matson et al., 2011; Sharma and Shaw, 2012). In 2006, The United States Food and Drug Administration approved risperidone for the treatment of irritability in ASD in children and adolescents 5–16 years of age. However, significant concerns about tolerability remain and suggest that benefits of this medication must be carefully weighed against the risks. Evidence from controlled studies of alpha-2 agonists for ADHD-related symptoms in ASD is inconsistent and response rates are relatively low. Open-label studies of guanfacine (Intuniv) appear promising but additional controlled studies are needed. A retrospective analysis (Posey et al., 2004) of 80 patients indicated reduction in hyperactivity and inattention among children with ASD and higher cognitive functioning. Alpha-2 agonists may be a reasonable alternative or augmentation strategy and have the advantage of being relatively benign. Amantadine may be considered in the treatment of ASD but its use for ADHD-related symptoms is not yet supported by research (Hosenbocus and Chahal, 2013). Other medications like melatonin, antioxidants, acetylcholinesterase inhibitors, and naltrexone, are currently considered off-label for ASD, but further randomized controlled trials are necessary (Rossignol, 2009).

For all medication categories except MPH, no studies are available on pre-school children.

## Psychosocial Interventions

An up to date literature survey on psychosocial interventions in children with both ADHD and ASD has not revealed any results. In a comprehensive review on the treatment of these co-occurring conditions Davis and Kollins (2012) mention that there are similarities across approaches to treat both disorders. In both, treatment uses conditioning procedures, which have evolved in time to draw on a social learning theory (Brookman-Frazee et al., 2006). Whereas both ADHD and ASD include behaviorally oriented parental intervention, the role of the family is conceptualized in a different way; for ADHD “parent training” involves teaching parents to manage the behaviors of their children, in ASD “parent education” places more emphasis on individualized treatments that provide parents with tools to promote their child’s (social) skill development. Davis and Kollins suggest that bridging between these two strategies might benefit those with comorbid disorders.

While many studies have shown the importance of combining medication and psychosocial interventions (mostly parental education) for children with ADHD, there are only a few studies on the combined medication and behavioral approach in ASD children. Aman et al. (2009) primarily targeted frequent tantrums, self-injury, and aggression in a trial of risperidone treatment and parent training, but the combined effects on hyperactivity were also examined. Results indicated that children who received the combined treatments had lower rates of aggression and greater reduction in hyperactivity (requiring lower risperidone dose), as compared with children who received medication only.

Studies on psychosocial interventions in pre-school children with co-occurring ASD and ADHD are lacking.

## Summary and Future Directions

Research specifically focusing on co-occurring ADHD and ASD has only recently evolved, being previously limited by the DSM-IV exclusion criteria. What we have so far learned is that both disorders frequently co-occur, and when they do, they cause greater morbidity, and create a more complicated clinical challenge. The new DSM-V, allowing for a dual diagnosis, will hopefully facilitate research, by eliminating the exclusion of many patients and allowing the study of broader phenotypes. Most recent research focuses on etiology and clinical presentations, with less direct work on treatment and early intervention protocols. Very few studies have looked at pre-school children presenting with both conditions, on the impact of early intervention in this age group and its effect on developmental trajectory. Furthermore, many of the above quoted studies have used parent and/or teacher rating scales to assess clinical profiles, rather than direct clinical diagnoses of the two co-existing disorders. In the future, as a dual diagnosis is “officially” possible, studies using direct clinical assessment of both comorbid diagnoses are essential.

Future research should tackle two major hypotheses regarding the frequent co-occurrence of ASD and ADHD. The first is that ADHD and Autism are distinct, yet overlapping disorders which may share some common etiology, probably genetic. The second hypothesis is that the co-occurrence of autistic symptoms and ADHD “stands alone” as a distinct clinical disorder, with a distinct etiology, and a different developmental trajectory. These two hypotheses should be examined by studying the developmental trajectories in co-occurring ASD and ADHD, by defining common co-morbidity profiles in both, and by understanding differences and similarities in social perception, motor functions and language, cognition, and EF in each disorder and in the co-occurring “phenotype.” Defining early “endophenotypes” as suggested by Rommelse et al. (e.g., heritable vulnerability traits that form a link between genes and observable symptoms, e.g., neuroimaging, neuropsychological functions) of both disorders, should serve to increase the chance of identifying genetic markers for each one and for both together (Cross-Disorder Group of the Psychiatric Genomics Consortium, 2013).

In 2010, Gillberg et al. have coined the ESSENCE concept (Gillberg, 2010) an acronym for early symptomatic syndromes eliciting neurodevelopmental clinical examinations, pointing to the fact that major problems in at least one developmental domain before age 5 years often signals major problems in the same or overlapping domains years later. They suggest “There is no time to wait,” implicating intervention should be the main goal, and not necessarily the categorical diagnosis. They go on to suggest intervention should be broad, addressing the multiple aspects of developmental disorders at this young age. Future studies should focus on early identification and intervention strategies in this specific “co-morbid” group, with an emphasis on pre-school children, using prospective design, even before pathophysiology is fully understood.

## Conflict of Interest Statement

The author declares that the research was conducted in the absence of any commercial or financial relationships that could be construed as a potential conflict of interest.

## References

[B1] Aman M. G. McDougle C. J. Scahill L. Handen B. Arnold L. E. Johnson C. (2009). Medication and parent training in children with pervasive developmental disorders and serious behavior problems: results from a randomized clinical trial. J. Am. Acad. Child Adolesc. Psychiatry 48, 1143–1154 10.1097/CHI.0b013e3181bfd669 19858761 PMC3142923

[B2] Andersen P. N. Hovik K. T. Skogli E. W. Egeland J. Oie M. (2013). Symptoms of ADHD in children with high-functioning autism are related to impaired verbal working memory and verbal delayed recall. PLoS ONE 8:e64842 10.1371/journal.pone.0064842 23717667 PMC3661504

[B3] Antshel K. M. Remer R. (2003). Social skills training in children with attention deficit hyperactivity disorder: a randomized-controlled clinical trial. J. Clin. Child Adolesc. Psychol. 32, 153–165 10.1207/15374420360533149 12611031

[B4] Arnold L. E. Aman M. G. Cook A. M. Witwer A. N. Hall K. L. Thompson S. (2006). Atomoxetine for hyperactivity in autism spectrum disorders: placebo-controlled crossover pilot trial. J. Am. Acad. Child Adolesc. Psychiatry 45, 1196–1205 10.1097/01.chi.0000231976.28719.2a 17003665

[B5] Arnold L. E. Aman M. G. Li X. Butter E. Humphries K. Scahill L. (2012). Research Units of Pediatric Psychopharmacology (RUPP) Autism Network randomized clinical trial of parent training and medication: one-year follow-up. J. Am. Acad. Child Adolesc. Psychiatry 51, 1173–1184 10.1016/j.jaac.2012.08.028 23101743 PMC3772659

[B6] Barkley R. A. DuPaul G. J. McMurray M. B. (1990). Comprehensive evaluation of attention deficit disorder with and without hyperactivity as defined by research criteria. J. Consult. Clin. Psychol. 58, 775–789 10.1037/0022-006X.58.6.775 2292627

[B7] Baron-Cohen S. Ring H. A. Wheelwright S. Bullmore E. T. Brammer M. J. Simmons A. (1999). Social intelligence in the normal and autistic brain: an fMRI study. Eur. J. Neurosci. 11, 1891–1898 10.1046/j.1460-9568.1999.00621.x 10336657

[B8] Biederman J. Newcorn J. Sprich S. (1991). Comorbidity of attention deficit hyperactivity disorder with conduct, depressive, anxiety, and other disorders. Am. J. Psychiatry 148, 564–577 2018156 10.1176/ajp.148.5.564

[B9] Bishop D. V. Baird G. (2001). Parent and teacher report of pragmatic aspects of communication: use of the children’s communication checklist in a clinical setting. Dev. Med. Child Neurol. 43, 809–818 10.1017/S0012162201001475 11769267

[B10] Brookman-Frazee L. Stahmer A. Baker-Ericzén M. J. Tsai K. (2006). Parenting interventions for children with autism spectrum and disruptive behavior disorders: opportunities for cross-fertilization. Clin. Child Fam. Psychol. Rev. 9, 181–200 10.1007/s10567-006-0010-4 17053963 PMC3510783

[B11] Buitelaar J. K. van der Wees M. Swaab-Barneveld H. van der Gaag R. J. (1999). Verbal memory and performance IQ predict theory of mind and emotion recognition ability in children with autistic spectrum disorders and in psychiatric control children. J. Child Psychol. Psychiatry 40, 869–881 10.1111/1469-7610.00505 10509882

[B12] Canitano R. Scandurra V. (2011). Psychopharmacology in autism: an update. Prog. Neuropsychopharmacol. Biol. Psychiatry 35, 18–28 10.1016/j.pnpbp.2010.10.015 21034789

[B13] Cantwell D. P. (1996). Attention deficit disorder: a review of the past 10 years. J. Am. Acad. Child Adolesc. Psychiatry 35, 978–987 10.1097/00004583-199608000-00008 8755794

[B14] Carlson C. L. Mann M. (2000). Attention-deficit/hyperactivity disorder, predominantly inattentive subtype. Child Adolesc. Psychiatr. Clin. N. Am. 9, 499–510, vi. 10944653

[B15] Carlsson L. H. Norrelgen F. Kjellmer L. Westerlund J. Gillberg C. Fernell E. (2013). Coexisting disorders and problems in preschool children with autism spectrum disorders. ScientificWorldJournal 2013, 213979 10.1155/2013/213979 23737708 PMC3655672

[B16] Cooper M. Martin J. Langley K. Hamshere M. Thapar A. (2014). Autistic traits in children with ADHD index clinical and cognitive problems. Eur. Child Adolesc. Psychiatry 23, 23–34 10.1007/s00787-013-0398-6 23616179 PMC3899449

[B17] Cross-Disorder Group of the Psychiatric Genomics Consortium. (2013). Genetic relationship between five psychiatric disorders estimated from genome-wide SNPs. Nat. Genet. 45, 984–994 10.1038/ng.2711 23933821 PMC3800159

[B18] Davis N. O. Kollins S. H. (2012). Treatment for co-occurring attention deficit/hyperactivity disorder and autism spectrum disorder. Neurotherapeutics 9, 518–530 10.1007/s13311-012-0126-9 22678458 PMC3441928

[B19] Di Martino A. Zuo X. N. Kelly C. Grzadzinski R. Mennes M. Schvarcz A. (2013). Shared and distinct intrinsic functional network centrality in autism and attention-deficit/hyperactivity disorder. Biol. Psychiatry 74, 623–632 10.1016/j.biopsych.2013.02.011 23541632 PMC4508007

[B20] Eiraldi R. B. Power T. J. Karustis J. L. Goldstein S. G. (2000). Assessing ADHD and comorbid disorders in children: the Child Behavior Checklist and the Devereux Scales of Mental Disorders. J. Clin. Child Psychol. 29, 3–16 10.1207/S15374424jccp2901_2 10693028

[B21] Flicek M. (1992). Social status of boys with both academic problems and attention-deficit hyperactivity disorder. J. Abnorm. Child Psychol. 20, 353–366 10.1007/BF00918981 1527277

[B22] Frazier T. W. Shattuck P. T. Narendorf S. C. Cooper B. P. Wagner M. Spitznagel E. L. (2011). Prevalence and correlates of psychotropic medication use in adolescents with an autism spectrum disorder with and without caregiver-reported attention-deficit/hyperactivity disorder. J. Child Adolesc. Psychopharmacol. 21, 571–579 10.1089/cap.2011.0057 22166171 PMC3279713

[B23] Friedman S. R. Rapport L. J. Lumley M. Tzelepis A. VanVoorhis A. Stettner L. (2003). Aspects of social and emotional competence in adult attention-deficit/hyperactivity disorder. Neuropsychology 17, 50–58 10.1037/0894-4105.17.1.50 12597073

[B24] Gadow K. D. DeVincent C. J. Pomeroy J. (2006). ADHD symptom subtypes in children with pervasive developmental disorder. J. Autism Dev. Disord. 36, 271–283 10.1007/s10803-005-0060-3 16477513

[B25] Gadow K. D. DeVincent C. J. Pomeroy J. Azizian A. (2004). Psychiatric symptoms in preschool children with PDD and clinic and comparison samples. J. Autism Dev. Disord. 34, 379–393 10.1023/B:JADD.0000037415.21458.93 15449514

[B26] Gargaro B. A. Rinehart N. J. Bradshaw J. L. Tonge B. J. Sheppard D. M. (2011). Autism and ADHD: how far have we come in the comorbidity debate? Neurosci. Biobehav. Rev. 35, 1081–1098 10.1016/j.neubiorev.2010.11.002 21093480

[B27] Geurts H. M. Ridderinkhof K. R. Scholte H. S. (2013). The relationship between grey-matter and ASD and ADHD traits in typical adults. J. Autism Dev. Disord. 43, 1630–1641 10.1007/s10803-012-1708-4 23138728

[B28] Gillberg C. (2010). The ESSENCE in child psychiatry: early symptomatic syndromes eliciting neurodevelopmental clinical examinations. Res. Dev. Disabil. 31, 1543–1551 10.1016/j.ridd.2010.06.002 20634041

[B29] Greene R. W. Biederman J. Faraone S. V. Ouellette C. A. Penn C. Griffin S. M. (1996). Toward a new psychometric definition of social disability in children with attention-deficit hyperactivity disorder. J. Am. Acad. Child Adolesc. Psychiatry 35, 571–578 10.1097/00004583-199605000-00011 8935203

[B30] Grzadzinski R. Di Martini A. Brady E. Mairena M. A. O’Neale M. Petrova E. (2011). Examining autistic traits in children with ADHD: does the autism spectrum extend to ADHD? J. Autism Dev. Disord. 41, 1178–1191 10.1007/s10803-010-1135-3 21108041 PMC3123401

[B31] Guttmann-Steinmetz S. Gadow K. D. Devincent C. J. (2009). Oppositional defiant and conduct disorder behaviors in boys with autism spectrum disorder with and without attention-deficit hyperactivity disorder versus several comparison samples. J. Autism Dev. Disord. 39, 976–985 10.1007/s10803-009-0706-7 19288296

[B32] Hodgens J. B. Cole J. Boldizar J. (2000). Peer-based differences among boys with ADHD. J. Clin. Child Psychol. 29, 443–452 10.1207/S15374424JCCP2903_15 10969428

[B33] Holtmann M. Bölte S. Poustka F. (2007). Autism spectrum disorders: sex differences in autistic behaviour domains and coexisting psychopathology. Dev. Med. Child Neurol. 49, 361–366 10.1111/j.1469-8749.2007.00361.x 17489810

[B34] Hosenbocus S. Chahal R. (2013). Amantadine: a review of use in child and adolescent psychiatry. J. Can. Acad. Child Adolesc. Psychiatry 22, 55–60 23390434 PMC3565716

[B35] Hoza B. Mrug S. Gerdes A. C. Hinshaw S. P. Bukowski W. M. Gold J. A. (2005). What aspects of peer relationships are impaired in children with attention-deficit/hyperactivity disorder? J. Consult. Clin. Psychol. 73, 411–423 10.1037/0022-006X.73.3.411 15982139

[B36] Jahromi L. B. Kasari C. L. McCracken J. T. Lee L. S. Aman M. G. McDougle C. J. (2009). Positive effects of methylphenidate on social communication and self-regulation in children with pervasive developmental disorders and hyperactivity. J. Autism Dev. Disord. 39, 395–404 10.1007/s10803-008-0636-9 18752063 PMC4374624

[B37] Klassen A. F. Miller A. Fine S. (2004). Health-related quality of life in children and adolescents who have a diagnosis of attention-deficit/hyperactivity disorder. Pediatrics 114, e541–e547 10.1542/peds.2004-0844 15520087

[B38] Kotte A. Joshi G. Fried R. Uchida M. Spencer A. Woodworth K. Y. (2013). Autistic traits in children with and without ADHD. Pediatrics 132, e612–e622 10.1542/peds.2012-3947 23979086 PMC3876754

[B39] Lecavalier L. Gadow K. D. DeVincent C. J. Edwards M. C. (2009). Validation of DSM-IV model of psychiatric syndromes in children with autism spectrum disorders. J. Autism Dev. Disord. 39, 278–289 10.1007/s10803-008-0622-2 18654843

[B40] Lee D. O. Ousley O. Y. (2006). Attention-deficit hyperactivity disorder symptoms in a clinic sample of children and adolescents with pervasive developmental disorders. J. Child Adolesc. Psychopharmacol. 16, 737–746 10.1089/cap.2006.16.737 17201617

[B41] Leyfer O. T. Folstein S. E. Bacalman S. Davis N. O. Dinh E. Morgan J. (2006). Comorbid psychiatric disorders in children with autism: interview development and rates of disorders. J. Autism Dev. Disord. 36, 849–861 10.1007/s10803-006-0123-0 16845581

[B42] Marton I. Wiener J. Rogers M. Moore C. Tannock R. (2009). Empathy and social perspective taking in children with attention-deficit/hyperactivity disorder. J. Abnorm. Child Psychol. 37, 107–118 10.1007/s10802-008-9262-4 18712471

[B43] Matson J. L. Sipes M. Fodstad J. C. Fitzgerald M. E. (2011). Issues in the management of challenging behaviours of adults with autism spectrum disorder. CNS Drugs 25, 597–606 10.2165/11591700-000000000-00000 21699271

[B44] Mikami A. Y. (2010). The importance of friendship for youth with attention-deficit/hyperactivity disorder. Clin. Child Fam. Psychol. Rev. 13, 181–198 10.1007/s10567-010-0067-y 20490677 PMC2921569

[B45] Milich R. Landau S. Kilby G. Whitten P. (1982). Preschool peer perceptions of the behavior of hyperactive and aggressive children. J. Abnorm. Child Psychol. 10, 497–510 10.1007/BF00920750 7161441

[B46] MTA Cooperative Group. (2004). National Institute of Mental Health Multimodal Treatment Study of ADHD follow-up: 24-month outcomes of treatment strategies for attention-deficit/hyperactivity disorder. Pediatrics 113, 754–761 10.1542/peds.113.4.754 15060224

[B47] Mulligan A. Anney R. J. O’Regan M. Chen W. Butler L. Fitzgerald M. (2009). Autism symptoms in attention-deficit/hyperactivity disorder: a familial trait which correlates with conduct, oppositional defiant, language and motor disorders. J. Autism Dev. Disord. 39, 197–209 10.1007/s10803-008-0621-3 18642069

[B48] Murray M. J. (2010). Attention-deficit/hyperactivity disorder in the context of Autism spectrum disorders. Curr. Psychiatry Rep. 12, 382–388 10.1007/s11920-010-0145-3 20694583

[B49] Nijmeijer J. S. Minderaa R. B. Buitelaar J. K. Mulligan A. Hartman C. A. Hoekstra P. J. (2008). Attention-deficit/hyperactivity disorder and social dysfunctioning. Clin. Psychol. Rev. 28, 692–708 10.1016/j.cpr.2007.10.003 18036711

[B50] Owen R. Sikich L. Marcus R. N. Corey-Lisle P. Manos G. McQuade R. D. (2009). Aripiprazole in the treatment of irritability in children and adolescents with autistic disorder. Pediatrics 124, 1533–1540 10.1542/peds.2008-3782 19948625

[B51] Pelham W. E. Bender M. E. Caddell J. Booth S. Moorer S. H. (1985). Methylphenidate and children with attention deficit disorder. Dose effects on classroom academic and social behavior. Arch. Gen. Psychiatry 42, 948–952 10.1001/archpsyc.1985.01790330028003 3899046

[B52] Piven J. Arndt S. Bailey J. Andreasen N. (1996). Regional brain enlargement in autism: a magnetic resonance imaging study. J. Am. Acad. Child Adolesc. Psychiatry 35, 530–536 10.1097/00004583-199604000-00020 8919716

[B53] Posey D. J. Puntney J. I. Sasher T. M. Kem D. L. McDougle C. J. (2004). Guanfacine treatment of hyperactivity and inattention in pervasive developmental disorders: a retrospective analysis of 80 cases. J. Child Adolesc. Psychopharmacol. 14, 233–241 10.1089/1044546041649084 15319020

[B54] Posey D. J. Wiegand R. E. Wilkerson J. Maynard M. Stigler K. A. McDougle C. J. (2006). Open-label atomoxetine for attention-deficit/hyperactivity disorder symptoms associated with high-functioning pervasive developmental disorders. J. Child Adolesc. Psychopharmacol. 16, 599–610 10.1089/cap.2006.16.599 17069548

[B55] Rao P. A. Landa R. J. (2013). Association between severity of behavioral phenotype and comorbid attention deficithyperactivity disorder symptoms in children with autism spectrum disorders. Autism 18:272–280 10.1177/1362361312470494 23739542 PMC4103643

[B56] Reiersen A. M. Constantino J. N. Volk H. E. Todd R. D. (2007). Autistic traits in a population-based ADHD twin sample. J. Child Psychol. Psychiatry 48, 464–472 10.1111/j.1469-7610.2006.01720.x 17501727

[B57] Riddle M. A. Yershova K. Lazzaretto D. Paykina N. Yenokyan G. Greenhill L. (2013). Preschool attention deficit/hyperactivity disorder treatment study (PATS) 6-year follow-up. J. Am. Acad. Child Adolesc. Psychiatry 52, 264–278 10.1016/j.jaac.2012.12.007 23452683 PMC3660093

[B58] Rommelse N. N. Franke B. Geurts H. M. Hartman C. A. Buitelaar J. K. (2010). Shared heritability of attention-deficit/hyperactivity disorder and autism spectrum disorder. Eur. Child Adolesc. Psychiatry 19, 281–295 10.1007/s00787-010-0092-x 20148275 PMC2839489

[B59] Rommelse N. N. Geurts H. M. Franke B. Buitelaar J. K. Hartman C. A. (2011). A review on cognitive and brain endophenotypes that may be common in autism spectrum disorder and attention-deficit/hyperactivity disorder and facilitate the search for pleiotropic genes. Neurosci. Biobehav. Rev. 35, 1363–1396 10.1016/j.neubiorev.2011.02.015 21382410

[B60] Ronald A. Edelson L. R. Asherson P. Saudino K. J. (2010). Exploring the relationship between autistic-like traits and ADHD behaviors in early childhood: findings from a community twin study of 2-year olds. J. Abnorm. Child Psychol. 38, 185–196 10.1007/s10802-009-9366-5 19908138 PMC4096900

[B61] Rossignol D. A. (2009). Novel and emerging treatments for autism spectrum disorders: a systematic review. Ann. Clin. Psychiatry 21, 213–236 19917212

[B62] Santosh P. J. Baird G. Pityaratstian N. Tavare E. Gringras P. (2006). Impact of comorbid autism spectrum disorders on stimulant response in children with attention deficit hyperactivity disorder: a retrospective and prospective effectiveness study. Child Care Health Dev. 32, 575–583 10.1111/j.1365-2214.2006.00631.x 16919137

[B63] Santosh P. J. Mijovic A. (2004). Social impairment in hyperkinetic disorder – relationship to psychopathology and environmental stressors. Eur. Child Adolesc. Psychiatry 13, 141–150 10.1007/s00787-004-0372-4 15254841

[B64] Sharma A. Shaw S. R. (2012). Efficacy of risperidone in managing maladaptive behaviors for children with autistic spectrum disorder: a meta-analysis. J. Pediatr. Health Care 26, 291–299 10.1016/j.pedhc.2011.02.008 22726714

[B65] Sikora D. M. Vora P. Coury D. L. Rosenberg D. (2012). Attention deficit/hyperactivity disorder symptoms, adaptive functioning, and quality of life in children with autism spectrum disorder. Pediatrics 130(Suppl. 2), S91–S97 10.1542/peds.2012-0900G 23118259

[B66] Sinzig J. Morsch D. Lehmkuhl G. (2008). Do hyperactivity, impulsivity inattention have an impact on the ability of facial affect recognition in children with autism and ADHD? Eur. Child Adolesc. Psychiatry 17, 63–72 10.1007/s00787-007-0637-9 17896119

[B67] St Pourcain B. Mandy W. P. Heron J. Golding J. Davey Smith G. Skuse D. H. (2011). Links between co-occurring social-communication and hyperactive-inattentive trait trajectories. J. Am. Acad. Child Adolesc. Psychiatry 50, 892–902 10.1016/j.jaac.2011.05.015 21871371 PMC3163265

[B68] Subcommittee on Attention Deficit/Hyperactivity Disorder, Steering Committee on Quality Improvement and Management Wolraich M. Brown L. Brown R. T. DuPaul G. (2011). ADHD: clinical practice guideline for the diagnosis, evaluation, and treatment of attention-deficit/hyperactivity disorder in children and adolescents. Pediatrics 128, 1007–1022 10.1542/peds.2011-2654 22003063 PMC4500647

[B69] Uekermann J. Kraemer M. Abdel-Hamid M. Schimmelmann B. G. Hebebrand J. Daum I. (2010). Social cognition in attention-deficit hyperactivity disorder (ADHD). Neurosci. Biobehav. Rev. 34, 734–743 10.1016/j.neubiorev.2009.10.009 19857516

[B70] van Steijn D. J. Richards J. S. Oerlemans A. M. de Ruiter S. W. van Aken M. A. Franke B. (2012). The co-occurrence of autism spectrum disorder and attention-deficit/hyperactivity disorder symptoms in parents of children with ASD or ASD with ADHD. J. Child Psychol. Psychiatry 53, 954–963 10.1111/j.1469-7610.2012.02556.x 22537103

[B71] Vora P. Sikora D. (2011). Society for Developmental and Behavioral. San Antonio, TX: Pediatrics

[B72] Whalen C. K. Henker B. Hinshaw S. P. (1985). Cognitive-behavioral therapies for hyperactive children: premises, problems, and prospects. J. Abnorm. Child Psychol. 13, 391–409 10.1007/BF00912724 4045009

[B73] Williams K. Brignell A. Randall M. Silove N. Hazell P. (2013). Selective serotonin reuptake inhibitors (SSRIs) for autism spectrum disorders (ASD). Cochrane Database Syst. Rev. 8, 10.1002/14651858.CD004677.pub3 PMC11990048 23959778

[B74] Yerys B. E. Wallace G. L. Sokoloff J. L. Shook D. A. James J. D. Kenworthy L. (2009). Attention deficit/hyperactivity disorder symptoms moderate cognition and behavior in children with autism spectrum disorders. Autism Res. 2, 322–333 10.1002/aur.103 19998356 PMC3012375

[B75] Young S. Heptinstall E. Sonuga-Barke E. J. Chadwick O. Taylor E. (2005). The adolescent outcome of hyperactive girls: self-report of psychosocial status. J. Child Psychol. Psychiatry 46, 255–262 10.1111/j.1469-7610.2004.00350.x 15755302

